# Experimental Models of Cryptococcosis

**DOI:** 10.1155/2012/626745

**Published:** 2011-10-06

**Authors:** Wilber Sabiiti, Robin C. May, E. Rhiannon Pursall

**Affiliations:** School of Biosciences, University of Birmingham, Edgbaston, Birmingham B15 2TT, UK

## Abstract

Cryptococcosis is a life-threatening fungal disease that infects around one million people each year. Establishment and progression of disease involves a complex interplay between the fungus and a diverse range of host cell types. Over recent years, numerous cellular, tissue, and animal models have been exploited to probe this host-pathogen interaction. Here we review the range of experimental models that are available for cryptococcosis research and compare the relative advantages and limitations of the different systems.

## 1. Introduction

Cryptococcosis presents in three forms—cutaneous, pulmonary, and meningococcal—and is a life-threatening fungal disease. Although the genus *Cryptococcus* contains more than 50 species of free-living basidiomycete fungi, only two, *C. neoformans* and *C. gattii,* are significant pathogens of humans [1, 2]. Disease is typically caused in the immunocompromised, such as HIV/AIDS or organ transplant patients, and is usually attributable to *C. neoformans*. However, incidences of cryptococcosis in otherwise healthy individuals have been rising, and *C. gattii* is primarily responsible for these cases [2, 3]. Cryptococcal infection is acquired through inhalation of basidiospores or desiccated yeast cells into the lungs, from where cryptococci can potentially disseminate to all organs but with a predilection to the brain [1, 4–8]. Cryptococcal meningitis is estimated to kill 600,000 people annually worldwide, with more than 80% of deaths occurring in Sub-Saharan Africa [9].

In the course of infection through airways to lungs and from lungs to brain, *Cryptococcus* must overcome two major barriers: the innate and adaptive immune mechanisms of the host. The former consists of anatomical or physical barriers such as the mucosal or lung epithelium, the blood-brain barrier of the CNS, and phagocytic cells such as neutrophils, monocytes/macrophages, and dendritic cells. Successful evasion of the host defences results in cryptococcal colonization of host tissues and hence cryptococcosis. To better understand the pathogenesis of this disease, numerous *in vivo* and *in vitro* models have been developed to investigate features of cryptococcosis and address questions such as the course of cryptococcal infection, invasion of cellular barriers, and interactions with—and evasion of—the immune response. 

The aim of this review is to present all reported experimental models of Cryptococcosis and summarise recent and/or prolific discoveries using these. This will hopefully provide an evaluation of how different models can aid Cryptococcal research and give food for thought on how current and new models could be utilised in novel ways.

## 2. *In Vitro* Cellular Models

### 2.1. Monocytes and Macrophages

The role of monocytes and macrophages in cryptococcosis has been widely studied. Macrophages detect, phagocytose and kill extracellular microorganisms, and present antigen to T cells [10, 11]. These disease factors have been explored using *in vitro* models. 

**Figure 1:**
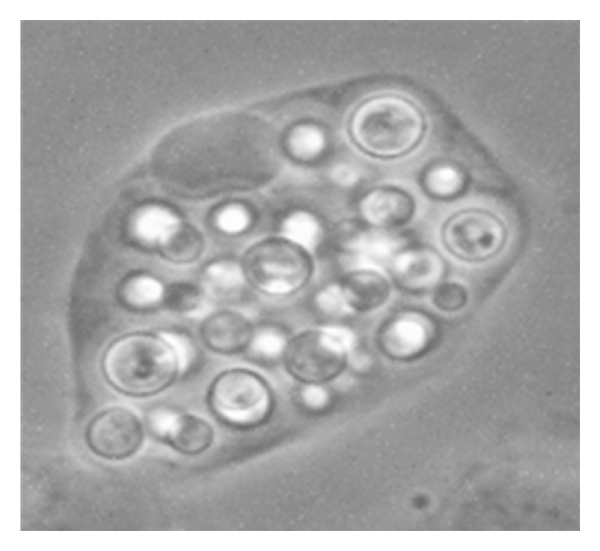
*Cryptococcus neoformans *can proliferate to a density of hundreds of yeast cells within macrophages. Here a J774 macrophage has been partially lysed to reveal intracellular *C. neoformans* after 18 hr of incubation. Image: W. Sabiiti.

Macrophage phagocytosis of nonopsonised cryptococcal cells is very poor, but dramatically enhanced by complement or immunoglobulin-based opsonins [12]. Dysfunctional phagocytic apparatus cripples the immune response; for example monocytes from HIV/AIDS patients that were unable to phagocytose cryptococci failed to induce lymphoproliferative response in a macrophage—lymphocyte coculture system [13]. Once *Cryptococcus* cells have been engulfed, macrophages can present Cryptococcal antigen and induce IL-1 expression and T-cell proliferation *in vitro* [14, 15]. However, cryptococci show a remarkable ability to survive and proliferate within macrophages, an adaptation that has been explored using both live imaging (Figure 1) [16–19] and gene expression [20, 21] approaches in macrophage cell lines (including J774 and RAW) and primary cells (including bone-marrow-derived murine cells and peripheral blood monocyte-derived human cells) [16, 17, 20, 22–24]. A noteworthy consideration when using *in vitro* models is that the *Cryptococcus*-cell interaction can differ depending on the type of macrophage used. For example, cryptococcal expulsion rates were found to be significantly higher in human primary macrophages compared to the J774 cell line [17]. However, despite the fact that different macrophage types are known to vary significantly in their behaviour *in vivo* [25], this aspect has yet to be extensively investigated in the context of cryptococcosis.

### 2.2. Dendritic Cells

Dendritic cells (DCs) constitute vital mediators of the initiation of adaptive immune response [26] and are regarded as professional antigen presenters. Although less well studied than macrophages, several aspects of DC function have been documented *in vitro*. Phagocytosis of live and heat-killed cryptococci and antigen presentation have been demonstrated in both human peripheral blood mononuclear cells (PBMCs), derived DCs and mouse bone-marrow-derived dendritic cells (BMDCs) [27, 28]. Internalization of live *C. neoformans* induced minimal TNF-alpha production by human monocyte-derived DCs and none in mouse-derived BMDCs, whereas human DCs incubated with acapsular cryptococci produced significantly higher amounts of TNF-alpha. This suggests that presence of capsule inhibits protective cytokine production. While it is clear from these studies that DCs can internalize and process both dead and live cryptococci, it is not known whether both dead and live antigen induce the same type and intensity of cytokine response. In addition, it is as yet undetermined whether intracellular cryptococci are eradicated by DCs or whether they survive, proliferate, and escape as seen in macrophages. 

### 2.3. Neutrophils

Neutrophils make up the largest population of phagocytes and are the first to be recruited to areas of infection. However their short lifespan means that they are a challenging cell type used in *in vitro* experimentation. Despite this limitation, investigations have shown that neutrophils are able to kill cryptococci *in vitro* [29, 30]. Although neutrophil cryptococci killing is typically considered to be mediated by the oxidative burst, neutrophils retain partial anticryptococcal function even when treated with respiratory burst inhibitors [31]. Unlike macrophages, neutrophils are also able to control extracellular cryptococci [32], although cryptococci produce mannitol, superoxide dismutase, and other ROS scavengers which help protect against extracellular killing by the respiratory burst [32, 33]. To date, however, it is not known whether cryptococci can persist to any degree within neutrophils, or how this interaction is modulated by the presence of macrophages at the infection site.

### 2.4. Eosonophils

Eosonophils, mostly associated with inflammatory response to helminthic parasites, have also been shown to be capable of phagocytosing and killing *C. neoformans* [34, 35]. *Cryptococcus*—eosonophil interactions have been studied using primary rat peritoneal eosinophils, in which it has been demonstrated that uptake of cryptococcal cells is strongly enhanced by antibody opsonization and is mediated by Fc*γ*RII and CD18 [34, 35]. Garro and colleagues further showed that coincubation of *C. neoformans*-stimulated eosinophils with CD4+ and CD8+ T cells from infected mice resulted in proliferation of T cells in an MHC 1- and 2-dependent manner, and hence eosinophils can act as antigen presenters and induce adaptive immune response to cryptococcal infection [35].

### 2.5. Lymphocytes

Lymphocytes, particularly CD4+ T cells, are important in the cell-mediated immune response to cryptococcosis [36]. Lymphocyte—*Cryptococcus* interaction models have used either primary mouse-derived or human PBMC-derived T lymphocytes and, to a lesser extent, immortalized T-cell lines. Studies using human lymphocytes have demonstrated a direct interaction between *C. neoformans* and lymphocytes. Both T cells and NK cells attach to *Cryptococcus* and directly inhibit its growth *in vitro* [36, 37]. Lymphocyte—*Cryptococcus* conjugate formation was enhanced when lymphocytes from mice immunized with heat killed *C. neoformans* were coincubated with cryptococcal yeast cells [38], suggesting that prior exposure activates this process, presumably through antigen processing and presentation by phagocytes to lymphocytes [39]. Intriguingly, both CD4+ and CD8+ T lymphocytes can kill extracellular cryptococci via granulysin, NK cells utilise perforin to achieve the same ends [40–43].

## 3. *In Vitro* Physical Barrier Models

### 3.1. Lung Epithelium

The inhalation route of infection makes the lung the first internal organ to be colonized by *Cryptococcus*. The interaction between *Cryptococcus* and epithelial cells lining the alveolar spaces is thus critical in regulating cryptococcal entry into the circulation system. The mechanisms that allow *Cryptococcus* to penetrate human alveolar cells remain largely uncharacterised. Initial investigation showed that glucuronoxylomannan is an important factor permitting the attachment of yeast to host cell and subsequent infiltration of, and damage to, the host intracellular environment [44]. *In vitro* experiments have also provided insight into how the capsule virulence factor promotes the ability of *Cryptococcus* to cause infection at the lung barrier. Human lung epithelial cells can internalize both encapsulated and acapsular strains of *C. neoformans* [45]. The lung surfactant protein-D (SP-D) was shown to bind to and promote phagocytosis of both encapsulated and acapsular *C. neoformans *by macrophages *in vitro* [46]. However, encapsulated cryptococci are not efficiently opsonized by SP-D and those that were phagocytosed resisted intracellular killing by macrophages [47]. In addition, the lung is the site of granuloma formation during pulmonary cryptococcosis (and potentially during latent infections). To date, however, no models have been established to address the role of lung epithelial cells in granuloma formation.

### 3.2. Blood-Brain Barrier

**Figure 2:**
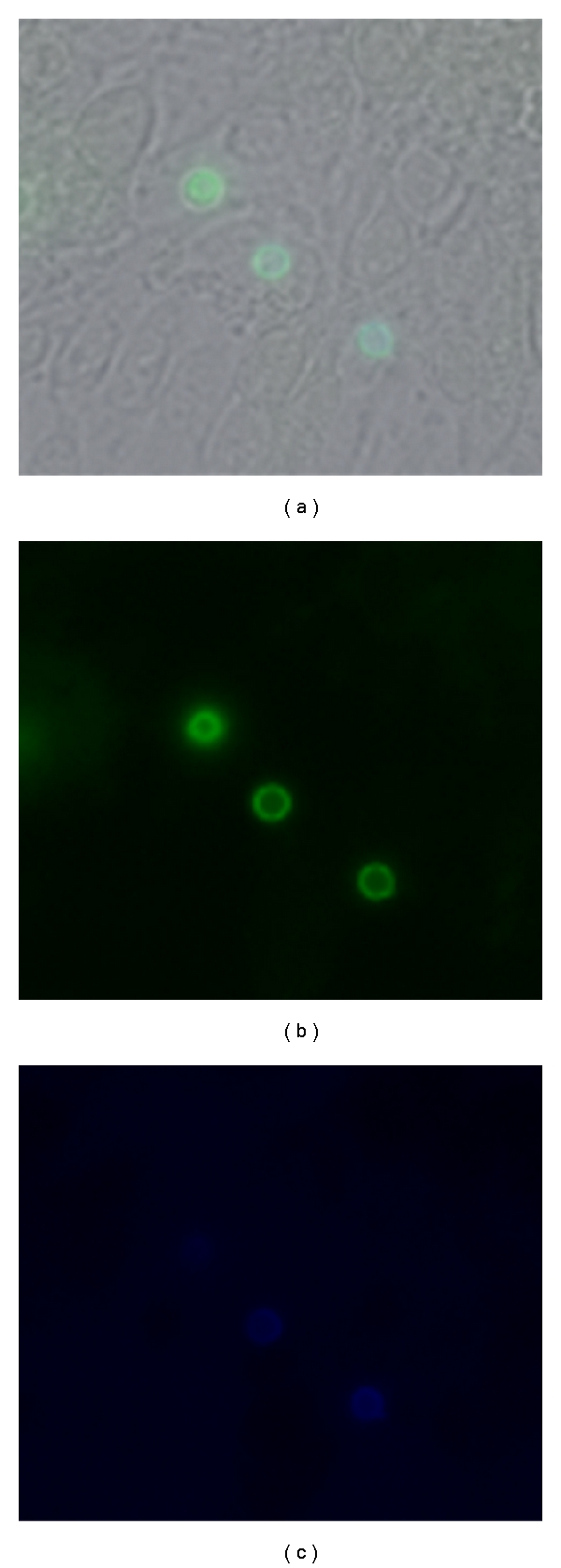
Fluorescence microscopy to determine adherent and internalized* Cryptococcus neoformans* on brain microvascular endothelial cells. *C. neoformans* serotype A strain H99 cells were prestained with FITC (green) before 4 hr incubation with brain endothelial cells and then counterlabelled with Calcofluor White (blue) to label extracellular adherent yeasts. (a) Merged image to reveal both endothelial cells and associated yeast cells, (b) and (c) Fluorescence images revealing the FITC and Calcofluor stained yeast cells, respectively.

Cryptococcal meningoencephalitis is the most devastating and fatal form of cryptococcosis. Modelling the blood-brain barrier (BBB) with the appropriate neurovascular properties has been a prominent challenge in cryptococcosis research. Isolation of human, rat and mouse brain microvascular endothelial cells (BMECs), which can be propagated *in vitro* to monolayers with BBB-like properties, has greatly improved the study of *Cryptococcus*—BBB interactions (Figure 2). Both human and mouse immortalized BMEC cell lines are commercially available and have been used in several studies, although freshly isolated BMECs are not so readily accessible.

*Cryptococcus* must penetrate the blood-brain barrier in order to cause meningococcal infection. Several studies using either primary or immortalized human, rat and mouse BMEC as BBB models have shown that BMEC can associate with and internalize yeast cells [39, 48–50]. Transwell chamber systems with BMEC monolayers have been used in some of the studies to demonstrate the occurrence of transcytosis across this cell layer [39, 50]. However *in vivo* the BBB is a complex tissue in which BMECs are supported by pericytes and astrocytes, a feature which cannot at present be recreated *in vitro*. In this context, the recent application of intravital imaging to examine cryptococcal traversal across the BBB *in vivo* represents a major advance for the field [51].

## 4. *In Vivo* Models

*In vivo* models can complement results of *in vitro* studies and also stand alone as valuable tools with which to make new discoveries and test out hypotheses. In this context, it is important to make the distinction between models that are used because they are vectors or carriers of human disease, and those that are used purely for their amenability in an experimental context. Those that are studied because they naturally harbour *Cryptococcus*, for example the koala [52], will not be discussed here. Instead we concentrate below on the range of model organisms exploited to undertake a whole-organism approach to studying *Cryptococcus*. In addition, the use of invertebrate models is an increasingly popular approach in many areas of biomedical research, including that of human fungal pathogens. We place a relatively large focus on these novel, emerging models over the more established and well-used vertebrate systems in order to highlight potential avenues for exploiting new model systems for cryptococcal research. 

### 4.1. Invertebrate Models

Invertebrates can be excellent models for disease. Advantages over their mammalian counterparts include reduced maintenance costs, fewer ethical restrictions, relatively short reproduction times, and large brood sizes. A key argument for the use of invertebrate models is that features of their immune systems allow rigorous investigation into human disease. Whereas vertebrates have evolved innate and adaptive immunity, invertebrates possess only the innate system, the most ancient form of pathogen defence. The basic underlying mechanisms of immune response can therefore be studied without potential confusion from adaptive immunity, which can be very species- or even individual-specific. 

A well-rehearsed argument that doubts the degree to which invertebrate models can be of value in biomedical research centres on the concept that the human immune system is vastly different from the invertebrate system. Indeed, adaptive immunity allows hosts to recognise molecular details of different invading pathogens, construct specific proteins to fight them, and maintain these proteins to protect against subsequent infections. However despite its lack of immune memory, the innate system of invertebrates is highly sophisticated and intricate and does not simply involve the same stereotypical response to every pathogen it encounters [53, 54]. Studies from organisms that use only innate immunity have demonstrated the complexity of this immune response [55, 56], and how it can vary according to a range of biotic and abiotic factors.

In addition, many human pathogens probably evolved their virulence mechanisms in more primitive organisms [57–60]. In the case of *Cryptococcus sp.*, these primitive hosts could have included free-living nematodes and amoebae. Adaptations that allowed the pathogens to escape natural invertebrate predators could have been translated into virulence factors for infecting mammalian hosts, and therefore studying interactions between the invertebrates and microbes could elucidate valuable information about disease. 

Invertebrates are also simple and efficient models in which to study fungal virulence factors, such as capsule growth and melanin production [59, 61]; morphological dimorphism [62]; phospholipase production [63]. Antifungal activity can also be tested rapidly and economically using invertebrate models, due to their amenability to high-throughput screens of chemical libraries that can measure key factors in drug development including host immune response, efficacy, and toxicity [64, 65].

#### 4.1.1. *Caenorhabditis elegans*

*Caenorhabditis elegans *has been employed for immunological study of many pathogens, and the past decade has seen it established as a model for *Cryptococcus neoformans* infection [61]. In this system a culture of *C. neoformans* in YPD liquid media is spread onto agar plates, usually with the addition of antibiotics, and incubated overnight. When *C. elegans* are transferred to these plates they acquire the pathogen orally, leading to fatal disease [66]. Critical similarities exist between pathogenicity in *C. elegans* and mammalian hosts, for example strains of *Cryptococcus* with reduced virulence in mammalian hosts generally show similar attenuation in *C. elegans* [61]. These parallels support the case for *C. elegans* constituting a sound model for investigating basic mechanisms underlying *Cryptococcus* infection, disease, and treatment. 

The molecular basis of infection and disease can be rigorously investigated in *C. elegans*, due to its amenability as a genetic model, and consequently a wealth of knowledge has been accumulated concerning immunity at the gene level. The potential to screen large numbers of mutant pathogens in *C. elegans* and identify genes important for virulence demonstrates a particular strength for the nematode model. A recent investigation identified mutations in *C. neoformans* that caused reduced survival in *ex vivo* cerebral spinal fluid and commensurate attenuation in *C. elegans* and the rabbit [67]. In addition, genes important for *C. neoformans* virulence in mammalian systems have been shown to be instrumental in causing death in *C. elegans* killing assays, highlighting a parallel between the immunity and pathogenesis of the *Cryptococcus*-human and *Cryptococcus*-worm model systems. For example, *C. neoformans* genes associated with virulence in both mammalian and nematode models include those associated with signal transduction pathways (GPA1, PKA1, PKR1, RAS1), laccase production (LAC1), and the {alpha} mating type [61]. 

*C. elegans* can use nonpathogenic forms of *Cryptococcus*, for example *C. laurentii* and *C. kuetzingii*, as a food source in the laboratory [23]. This lends support to the theory that the nematode is a natural predator of *Cryptococcus* and could therefore be an ideal model in which to investigate the evolution of virulence factors that have enabled certain strains of *Cryptococcus* to become pathogenic to worms and other hosts. 

However, a profound limitation of *C. elegans* as a model for investigating pathogenesis of mammalian disease is that the mode of infection is completely different to that in mammals. The nematode ingests the pathogen, and cryptococcal growth is restricted to the intestine, in marked contrast to the lung inhalation and subsequent dissemination that is characteristic of human infections. Along with the limitations associated with this entirely different mode of infection, it is not possible to administer an exact pathogen dose in this model, which could severely constrain the scope of certain experiments. In addition, phagocytes are not present in *C. elegans*, so in terms of studying phagocytosis in a whole organism model a more favourable invertebrate model may be an insect or amoeba.

#### 4.1.2. Amoeboid Models

Soil amoebae are environmental reservoirs of human pathogens [68] and therefore show promise as model hosts in the laboratory setting. Amoebae feed by phagocytosis of microorganisms, in a process that is essentially similar to the phagocytosis of microbes by human macrophages [69]. Amoebae therefore provide a simple model in which to investigate aspects of this fundamental immune response. Additionally, there are a number of characteristics that make these organisms valuable experimental models, primarily with respect to genetic tools.

**Acantamoeba castellanii**

In terms of using invertebrate hosts to study evolution of human pathogen virulence, the interaction between *Cryptococcus sp*. and *A. castellanii* provides interesting and unique opportunity for investigation. *Cryptococcus* can cause amoebae to lyse following phagocytosis [70], and a popular theory postulates that this adaptation to overcome predation could have been a precursor to the escape from mammalian phagocytes that cryptococci demonstrate *in vitro* [71]. In support of this, characteristics that promoted *Cryptococcus* survival in *A. castellanii* were also identified as virulence factors that enhanced *Cryptococcus* parasitism in macrophages [71]. Although *A. castellanii* may not be as genetically well characterised as the amoeboid model *D. discoideum*, the former has the advantage of remaining viable in laboratory conditions above 25°C, therefore more accurately simulating conditions of human infection, which occur in the critical 32–37°C window [72].

An important feature of the *A. castellanii* model is that this organism is killed by *Cryptococcus*. On a basic level this suggests that *Cryptococcus* readily acts as a pathogen in amoebae, which could confer an advantage over another invertebrate, such as *D. melanogaster*, which is not killed by injection of *Cryptococcus* and therefore may not be such a valuable a model of pathogenesis.

**Dictyostelium discoideum**

*Dictyostelium discoideum* has proved a useful tool for studying intracellular proliferation of human pathogens including *Cryptococcus.* The small haploid genome of *D. discoideum* has been sequenced [73], and is particularly amenable to genetic manipulation [68, 74].

Important parallels in terms of cryptococcal virulence are present between *D. discoideum* and human hosts. For example the fungal capsule is important for *C. neoformans* infection of *D. discoideum*, as acapsular mutants did not replicate in the amoebae [59]. Another important finding in *D. discoideum* showed that *C. neoformans* caused disease with increased efficiency following growth in the amoeboid model, indicating that adaptations for survival in the host translated into virulence factors [59].

#### 4.1.3. Insect Models

After pathogen recognition, the insect immune response follows a characteristic sequence of events involving two major classes of effector systems—cellular and humoral [75, 76]. The humoral immune response concerns the production of antimicrobial peptides and induction of enzyme cascades which function to minimise harm caused by the pathogen. Circulating haemocytes within the insect haemocoel produce the cellular response and rapidly fight the pathogen using three key cellular defences: phagocytosis, nodule formation, and encapsulation [77, 78]. Assays to measure insect immune response can be applied to a variety of species and employed in a high-throughput manner. For example, *in vivo* phagocytosis can be assessed by nondestructive extraction of haemolymph containing phagocytosed pathogens from insects and then analysed using *in vitro* assays. Given the importance of phagocytosis in cryptococcosis research, insects may therefore prove a valuable experimental tool.

Despite the many advantages of insect models for human disease research, there are a number of important limitations. Insects lack most organs found in humans, such as lungs, kidneys, and hearts. Given that organs such as the lungs are particularly important in *Cryptococcus* infection and disease, this highlights an obvious limitation of insects rather than mammalian models for this fungal pathogen. In a similar vein, the blood-brain barrier is a particularly important area of investigation for *Cryptococcus* research, but the lack of blood capillaries in the insect body means that this aspect of the disease cannot be studied in insect hosts.

**Galleria mellonella**

The larvae of the greater wax moth, *Galleria mellonella*, have been used as whole-organism virulence models for various species of pathogenic fungi. This organism can live at mammalian body temperature, thereby allowing temperature-sensitive investigation of pathogenesis. The larva also provides a good infection model because it is easy to inoculate with specific doses of fungal pathogen, owing largely to its size (approximately 1.5–2.5 cm in length), which contrasts with the smaller size of insects such as *Drosophila*, in which controlling dose per insect is challenging [79]. To date, the *G. mellonella* model has primarily been used to assess virulence and/or antifungal activity. Inoculation of microbes into the haemocoel is minimally invasive because piercing of the haemocoel is not required. Instead applying gentle pressure can open up the base of the proleg, and a needle is inserted. The aperture reseals on the release of pressure [79]. This nonpiercing method means that immune response directed at wound repair does not affect the results of experiments, which would be the case if insects were injected through the cuticle.

*G. mellonella* has been advocated as a particularly good model for diseases that disseminate through the body via the bloodstream, such as *Cryptococcus neoformans* and *Candida albicans* [79]. The development of *G. mellonella* as a model for *Cryptococcus neoformans* was first achieved by Mylonakis and colleagues in 2005 [80]. All *C. neoformans* strains tested caused death of the insect host, despite effective phagocytosis by insect haemocytes [80]. This suggests that *Cryptococcus* virulence may rely on the same mechanisms in *G. mellonella* as it does in *in vitro* assays, during which the phagocytic immune response is evaded and even exploited by the pathogen.

Recently the *G. mellonella* system was employed to investigate whether the antifungal activity of Fluconazole could be enhanced with the addition of other drugs. Fluconazole proved to have greater beneficial effect for *G. mellonella* survival when administered in combination with the antihistamine Astemizole and a closely related analog (A2) [81]. The results of this *in vivo* study were supported and enhanced by an *in vitro* experiment, in which the usually fungistatic Fluconazole became fungicidal when combined with Astemizole and A2 [81]. This investigation demonstrates how survival data from an insect model that is relatively easy to infect and monitor in a high-throughput manner can support *in vitro* mechanistic discovery.

**Drosophila melanogaster**

Infection of *D. melanogaster* with *C. neoformans* can be achieved by a variety of methods, and both dissemination of disease and local infection can be investigated. Injection into the haemocoel is done using a small needle inserted into the thorax or abdomen (e.g., [23]). This represents a systemic infection because the pathogen is administered directly into the haemolymph. A limitation of this method is that it is far removed from the natural pathway of infection, as microorganisms such as *Cryptococcus* would not be able to cross the insect cuticle unless an opening was already present. An alternative method is to administer the pathogen by incorporating it into the fly food.

Interest in *D. melanogaster* as a model of human disease has grown in recent years, with the continuing realisation that immune signalling pathways are highly conserved between fly and human [82, 83]. Of particular relevance to fungal disease is the finding that Toll receptor activation downstream of fungal infection leads to the production of antifungal peptides by *D. melanogaster* [84].

Wild-type *D. melanogaster* shows resistance to fungal pathogens administered by injection into the haemocoel including *Candida albicans*, *Aspergillus fumigatus*, and *Cryptococcus neoformans*. Experiments have shown that the Toll pathway is crucial in this resistance [84, 85]. In contrast, the Toll pathway is not involved in pathogen defence when the method of *Cryptococcus neoformans* infection is ingestion [85]. This research using *Drosophila* has suggested that *Cryptococcus neoformans* may elicit different responses in the systemic- and digestive-related immunity, in this host at least [85].

### 4.2. Vertebrate Models

For certain elements of fungal pathogen research, the benefit of the adaptive immune system of vertebrates will always be a feature that renders them more valuable as models than invertebrates. Vertebrate models have been extensively covered in other reviews (e.g., [86, 87]) but are discussed briefly below.

#### 4.2.1. Mouse (*Mus musculus*)

The majority of *Cryptococcus*-related work utilising mammalian systems has been carried out using mice. Due to the fact that murine models are well established as valuable study systems in many research areas including fungal pathogenesis, an in-depth discussion of mice in *Cryptococcus* research is not within the scope of the present review. Briefly, however, mouse models are a popular choice because they are well established and characterised in medical research, and a variety of genetic backgrounds are available. Infection with *Cryptococcus* can be achieved by a variety of methods, including intranasally, intraperitoneally, intracerebrally, intravenously, intratracheally, and via inhalation [88, 89], which opens up a range of experimental opportunities. In addition, the vast array of information available on the mouse immune system means that parallels and pitfalls can be readily identified, allowing for the design of highly refined experiments.

#### 4.2.2. Rat (*Rattus rattus*)

The rat model is comparable to mouse, but with a few advantages. The slightly larger body size allows a variety of experimental manipulations to be achieved with relative ease, including endotracheal intubation, bronchoalveolar lavage, serial venepuncture, CSF collection, radiography, computed tomography (CT), and magnetic resonance imaging (MRI) [88]. The rat has also been reported to develop chronic pulmonary cryptococcosis in the wild [90], suggesting this organism as a potentially valuable disease model. A model mimicking pulmonary cryptococcosis establishment, induced by *C. gattii *intratracheal inoculation in immunocompetent rat hosts, was recently established [88]. This opens up the opportunity to use this model to study more complex elements of cryptococcal etiology, such as long-term latency or the emergence of *C. gattii* as a primary pathogen. The rat model has been very useful in research into mammalian host responses to *Cryptococcus*. For example expression of acidic mammalian chitinase (AMCase) in response to infection by *Cryptococcus*, which contains chitin in its cell wall, could be a potential mediator of asthma [91].

#### 4.2.3. Guinea Pig (*Cavia porcellus*)

The Guinea pig model was first established for cryptococcal disease relatively recently by Kirkpatrick et al. [92]. The larger size of Guinea pigs compared to mice means they are ideal for more sensitive experimental manipulation. For example, intravenous inoculation can be relatively easily achieved in Guinea pigs. Another benefit of the Guinea pig model for fungal research is that oral doses of antifungals sufficient to control fungal infections are similar to the doses in humans [93]. An example of the utilisation of the Guinea pig model is the investigation into the effectiveness of an intravenous delivery of the antifungal itraconazole in fighting disseminated fungal infections including cryptococcosis [93]. 

#### 4.2.4. Rabbit (*Oryctolagus cuniculus*)

The rabbit has been advocated as a model for cryptococcal meningitis mainly due to its size in comparison to other mammalian study systems. For example, Steen et al. [94] chose the rabbit model because its relatively large body size enables the study of yeast at the site of infection, which is more difficult in smaller mammals such as mice and Guinea pigs. Historically the rabbit model was not an appealing choice for cryptococcal disease research because the organism appeared to be naturally resistant to this pathogen [95]. However the potential for this model increased when pretreatment with corticosteroids and subsequent *Cryptococcus* inoculation successfully resulted in the development of chronic cryptococcal meningitis [95]. A recent utilisation of this model involved measuring survival of *Cryptococcus* mutants in rabbits to establish whether mutations causing reduced attenuation in cerebral spinal fluid showed corresponding reduced virulence *in vivo* [67]. This is an example of the use of a whole-organism model to validate hypotheses of disease attenuation developed from *in vitro* experiments.

#### 4.2.5. Zebrafish (*Danio rerio*)

The zebrafish is emerging as an attractive model system for a variety of human diseases. Mutagenesis and screening can be done on a large scale with the zebrafish, which is a fairly unique feature in vertebrate models. Another benefit to this model is that it can be maintained at relatively low cost, with few ethical restraints. Thus the zebrafish in many ways has the economical advantages of an invertebrate model whilst also possessing all of the vertebrate immune system features that researchers in fungal pathogenesis require. 

**Figure 3:**
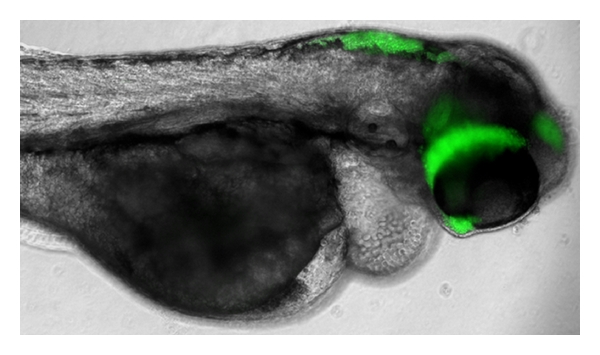
Zebrafish embryo 48 hours after infection with *Cryptococcus neoformans* strain H99 expressing GFP. Image Courtesy of S. A. Johnston, University of Birmingham, UK. GFP-expressing yeast was developed by Voelz et al. [99].

The zebrafish has not yet been used to investigate infection and disease in relation to *Cryptococcus*—however this application is under development (Figure 3). This model has been used for other human fungal pathogens, lending hope to the idea that a disease model for *Cryptococcus* could soon be developed. For example, a comprehensive infection model of the zebrafish with *Candida albicans* has been established, in which pathogen morphogenesis and gene expression, and host immune response, were monitored [96]. Fish were killed by *C. albicans* in a dose-dependent manner, and infection was established at a number of different sites, indicating that this organism could be a valuable tool in fungal research.

## 5. *Ex Vivo* Models

*Ex vivo* organ culture could be considered a way of achieving a balance between the advantages of *in vitro* and *in vivo* experimental models. The use of perfused organs provides a relatively authentic physiological environment and permits the study of pathogens crossing membranes, disseminating through host tissue, and migrating to the bloodstream—processes not captured by *in vitro* experimentation. Organs from higher animals, which would otherwise carry enormous ethical and economic implications, can be studied when perfused organs are chosen over whole animal models. For example, haemoperfused liver from pigs was recently established as a model for *C. albicans* investigation [97]. This model could be adapted for the study of other human fungal pathogens including *Cryptococcus*. An *ex vivo* model has also been established for swine trachea [98], and since *Cryptococcus* infects the lungs, this model could be of particular interest to adapt for investigation of this pathogen.

## 6. Closing Remarks

Cryptococcal research utilises a diverse range of experimental model systems, spanning from individual cells to unicellular whole organisms to mammalian models. Progress from hereon may benefit from different research disciplines exchanging knowledge and skills so that models already established in one field, such as drug development, can be adapted for another area of research such as pathogen evolution.
